# Nucleotide/Nucleoside Reverse Transcriptase Inhibitor‐Based HIV cART Regimens Produce Mixed Metabolic Responses in Diet‐Induced Prediabetic and Nonprediabetic Sprague Dawley Rats

**DOI:** 10.1155/bmri/9557266

**Published:** 2026-11-10

**Authors:** Amogelang Sedibe, Khanyisa Maswanganyi, Ntethelelo Sibiya, Trevor Nyakudya, Mlindeli Gamede

**Affiliations:** ^1^ Department of Physiology Faculty of Health Sciences University of Pretoria Pretoria Gauteng South Africa up.ac.za; ^2^ Pharmacology Division Faculty of Pharmacy Rhodes University Makhanda Eastern Cape South Africa ru.ac.za

**Keywords:** drug-metabolic complications, HIV-antiretroviral therapy, HIV-fixed-dose combination therapy, pancreatic health outcomes, prediabetes, Type 2 diabetes mellitus, Western lifestyle

## Abstract

**Background:**

The rise in HIV prevalence has led to more individuals using antiretroviral therapy (ART). These drugs, together with concurrent lifestyle factors, contribute to metabolic dysfunction, such as an increased risk of prediabetes. The asymptomatic nature of prediabetes may increase the probability of initiating ART without appropriate individualised regimen selection or required metabolic monitoring.

**Objectives:**

This study evaluated the effects of NRTI‐ and NNRTI‐based fixed‐dose combination regimens used as pre‐exposure prophylaxis (Truvada) and for HIV management (Triovir) on selected markers of glucose homeostasis.

**Methods:**

Forty‐two male Sprague Dawley rats (150–180 g) were randomly allocated into two main groups: nonprediabetic (NPD) (*n* = 21) and prediabetic (PD) (*n* = 21). Prediabetes was induced using a high‐fat, high‐carbohydrate (HFHC) diet over 20 weeks, followed by a treatment period lasting 12 weeks. Treatment procedure: the NPD and PD rats were further randomly subdivided into their respective ART regimens (*n* = 7/group). After the treatment period, the study was terminated, and tissues and organs of interest were collected for biochemical analyses.

**Results:**

HFHC feeding effectively induced a PD phenotype, characterised by elevated fasting glucose, glucose intolerance, increased homeostasis model assessment of insulin resistance (HOMA‐IR), hypertriglyceridaemia and weight gain (*p* < 0.05). Treatment with Truvada and Triovir attenuated these metabolic markers in PD and NPD groups. However, HOMA‐IR was elevated in NPD animals following both treatments. The administration of either regimen in NPD animals increased plasma C‐peptide levels, whereas plasma glucagon remained unchanged. In PD animals, Truvada treatment increased C‐peptide concentrations, whereas Triovir reduced them. Hepatic glucose exhibited a pattern comparable with the C‐peptide response in NPD animals. However, in PD groups, both treatments lowered glucagon levels and elevated hepatic glucose concentrations.

**Conclusion:**

Truvada and Triovir caused mixed responses on metabolic dysfunction in diet‐induced PD rats. These findings highlight the complex metabolic interactions of ART, warranting further mechanistic studies, particularly in populations on ART with pre‐existing metabolic risk.

## 1. Introduction

Glucose homeostasis is tightly regulated on a minute‐to‐minute basis as the human body cycles between fed and fasted states [1]. This physiological process is primarily controlled by two glucoregulatory hormones, insulin and glucagon, which are secreted by the pancreas islets [2]. These hormones act antagonistically, forming opposing reflex loops: Insulin decreases blood glucose levels by promoting glucose uptake in skeletal muscle and adipose tissue, enhancing hepatic glycogenesis and suppressing hepatic glucose production, whereas glucagon raises blood glucose levels by stimulating hepatic glycogenolysis and gluconeogenesis [2, 3]. Disruptions to this tightly regulated balance of hormones and glucose metabolic pathways result in elevated blood glucose levels that do not yet reach diabetic diagnostic thresholds, a metabolic condition known as prediabetes [4, 5].

In 2021, the global prevalence of prediabetes was 9.1% (464 million) based on impaired glucose tolerance (IGT) and 5.8% (298 million) based on impaired fasting glucose (IFG). Both are projected to rise by 2045, reaching 10.0% (638 million) for IGT and 6.5% (414 million) for IFG [6]. Prediabetes carries an annual conversion rate to Type 2 diabetes mellitus (T2DM) of approximately 5%–10% [4]. If not timely managed, prediabetes often progresses to overt T2DM, a recognised global epidemic impacting approximately 830 million individuals worldwide according to the World Health Organisation in 2022 and one of the leading contributors to global mortality [7]. The rising prevalence of prediabetes and its conversion to T2DM place increasing burdens on public health systems worldwide, necessitating focused efforts on intensified prevention programmes to reverse this high‐risk state. However, prediabetes remains an underexplored condition particularly in people living with HIV (PLHIV).

The PLHIV are particularly predisposed to developing prediabetes and diabetes [8–11]. This increasing dual burden of HIV and noncommunicable diseases is driven by ongoing urbanisation, which fosters modifiable risk factors such as excessive consumption of high‐calorie diets and sedentary lifestyles [12, 13]. Additionally, population ageing, resulting from improved effectiveness of combination antiretroviral therapy (cART), and chronic cART‐related toxicity further contribute to this trend [14]. Prolonged exposure to cART regimens has been associated with the development of hyperglycaemia and insulin resistance [11, 15]. Insulin resistance and pancreatic *β*‐cell dysfunction are key metabolic disturbances underlying prediabetes [4]. Insulin resistance impairs glucose uptake in insulin‐sensitive tissues, whereas *β*‐cell dysfunction reduces insulin secretion, together disrupting glucose homeostasis [16].

This compromised insulin action and secretion lead to inadequate suppression of hepatic glucose production during fasting and impaired clearance of postprandial glucose. Furthermore, abnormal glucagon secretion by pancreatic *α*‐cells may exacerbate hyperglycaemia by increasing hepatic glucose output [17]. Sustained hyperglycaemia during this state has been associated with the non‐enzymatic binding of glucose to haemoglobin, resulting in glycated haemoglobin (HbA1c) formation and fluctuating impairment in glucose tolerance [18]. Thus, prediabetic (PD) individuals may present with IFG and/or IGT or HbA1c levels ranging between 5.7% and 6.4% [5, 19]. Nucleoside/nucleotide reverse transcriptase inhibitors (NRTIs/NtRTIs) and non‐nucleoside reverse transcriptase inhibitors (NNRTIs) remain key components of current standard HIV treatment and prevention regimens. Tenofovir disoproxil fumarate (TDF), commonly included in first‐line NNRTI‐based cART regimens, remains widely used in resource‐limited settings [20]. TDF‐based regimens have been linked to weight loss and more favourable metabolic profiles [21]. However, the contributory role of TDF‐based regimens in dysglycaemia remains poorly elucidated.

Although clinical and epidemiological studies have established an association between prolonged cART exposure and dysglycaemia in PLHIV, the differential, mechanism‐level contribution of specific fixed‐dose regimens remains unresolved. In particular, no study to our knowledge has directly compared a PrEP‐context regimen (TDF/FTC) against a treatment‐context regimen (TDF/FTC/EFV) under matched nonprediabetic (NPD) and diet‐induced PD conditions within the same experimental design, nor examined a combined hormonal panel (insulin, C‐peptide, glucagon, HbA1c and hepatic glucose) that can distinguish altered insulin secretion from altered insulin sensitivity or clearance. Therefore, this study investigated the metabolic effects of TDF‐containing regimens, specifically TDF/emtricitabine/efavirenz (TDF/FTC/EFV) used for HIV management and TDF/FTC used for pre‐exposure prophylaxis, in the context of prediabetes.

## 2. Materials and Methods

### 2.1. Chemicals and Reagents

All chemicals and reagents were purchased from the following standard commercial suppliers: Fixed‐dose combination antiretroviral (ARV) drug tablets, Truvada and Triovir, were manufactured by Mylan laboratories Ltd. (Maharashtra, India) and obtained from BIOCOM Africa (Centurion, South Africa). Immunoassay kits from ABclonal Technology were also sourced from BIOCOM Africa. ACCU‐CHEK glucose strips and glucometers were obtained from Roche Diagnostics (United States). The standard rat chow was sourced from EPOL (Pretoria West, South Africa), the high‐fat, high‐carbohydrate diet from AVI‐Products (Pty) Ltd. (Cato Ridge, South Africa) and crystalline fructose from Tongaat Hulett (KwaZulu‐Natal, South Africa).

### 2.2. Ethical Clearance and Study Site

Ethical approval was obtained from both the Animal and Research Ethics Committee (AREC) of the University of Pretoria (Ethical Clearance Number: 134/2024) and the University of KwaZulu‐Natal (Ethical Clearance Number: AREC/00006018/2023(00021822). All in vivo procedures were conducted at the Biomedical Research Unit (BRU), University of KwaZulu‐Natal, following AREC guidelines.

### 2.3. Animal Husbandry

The housing of animals in this study was conducted in accordance with the *Guide for the Care and Use of Laboratory Animals*. The study utilised 42 male Sprague Dawley (SD) rats, weighing 150–180 g at the start of the experiment. Animals were bred and housed in the BRU at the University of KwaZulu‐Natal under standard laboratory conditions, including a temperature of 22^°^C ± 2^°^C, relative humidity of 55*%* ± 5*%* and a 12:12‐hour light‐dark cycle (lights on at 7:00 AM). The rats were group‐housed in conventional European standard Type 4 Cages fitted with stainless steel wire lids, enriched with red‐tinted polycarbonate tunnels and provided with dry wood shavings as bedding, which was changed biweekly. The rats had ad libitum access to standard rat chow and water during a 2‐week acclimatisation period prior to the initiation of the study phases. Only male rats were used in this study, consistent with our group′s previously validated diet‐induced prediabetes model and to avoid the additional metabolic variability introduced by the oestrous cycle.

### 2.4. Study Design

The study was conducted in two phases (Figure 1). The first phase involved the induction of prediabetes through the ad libitum administration of an HFHC (30% fat, 15% proteins and 55% carbohydrates) diet and drinking water supplemented with fructose (18%) over 20 weeks. The second phase involved the daily administration of cART drugs (Triovir and Truvada) for 12 weeks to mimic long‐term clinical use and evaluate potential metabolic complications associated with prolonged cART drug exposure.

**Figure 1:**
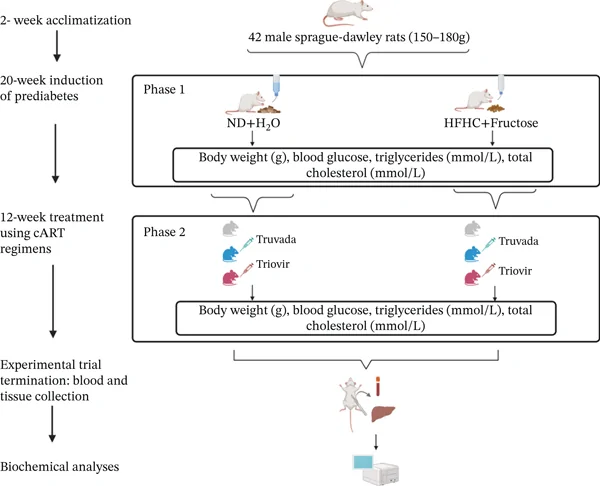
A schematic representation of the study design. A total of 42 male Sprague Dawley rats were used in this study. Briefly, the rats were randomly allocated into two dietary groups (*n* = 21/group): normal diet (ND) and high‐fat, high‐carbohydrates (HFHC) diet with fructose (18%) in drinking water to induce prediabetes over 20 weeks. Following induction, each dietary group was randomly further subdivided into three treatment arms (*n* = 7/group): no treatment (control), Truvada treatment or Triovir treatment, administered daily for 12 weeks to assess metabolic effects of long‐term cART. Throughout the experimental phases at specified intervals, key metabolic parameters were monitored. All animals were anaesthetized and tissue samples were collected for biochemical analyses.

#### 2.4.1. Phase 1: Induction of Prediabetes

Following acclimatisation, 42 male SD rats (150–180 g) were randomly assigned to two primary dietary groups (*n* = 21/group):

1. normal diet group, which received standard rat chow and water, and
2. HFHC diet group, which received an HFHC diet together with 18% fructose solution to induce prediabetes.

The respective dietary regimens were provided ad libitum and maintained throughout the study, including the treatment phase, to sustain the induced metabolic phenotype during pharmacological treatment. During this induction period, key metabolic parameters including body weight, plasma circulating metabolites (triglycerides and total cholesterol) and glucose tolerance were measured at baseline (Week 0), midpoint (Week 10) and endpoint (Week 20). At Week 20, prediabetes was confirmed through assessment of fasting plasma glucose (FPG), 2‐hour plasma glucose (2‐hr PG) and triglycerides. However, this study reports only Week 20 of the induction period, as explained in Section 3.1 and depicted in Table 1.

**Table 1:** FPG, 2‐h PG, body weight and triglycerides measured at Week 20 of the induction phase.

Variable	Nonprediabetic	Prediabetic
Fasting plasma glucose (mmol/L)	4.457 ± 0.232	6.3 ± 0.381
2‐hour plasma glucose (mmol/L)	6.271 ± 0.33	5.686 ± 0.241
Body weight (g)	454.6 ± 9.94	496.3 ± 29.57^∗^
Triglycerides (mmol/L)	1.483 ± 0.096	3.316 ± 0.619^∗^

*Note:* Values are presented as mean ± SEM (*n* = 5 per group).

^∗^ *p* < 0.05 denotes comparison with NPD group.

#### 2.4.2. Phase 2: cART Drug Treatment

After confirmation of the metabolic state achieved during Phase 1, each primary dietary group (*n* = 21) was further subdivided and randomly allocated into three treatment subgroups (*n* = 7/group), resulting in six final experimental groups (a 2 × 3 factorial design, comprising two metabolic states—NPD and PD, each subdivided into three treatment arms):

1. NPD untreated control (NPD)
2. NPD treated with Truvada (NPD + TRU)
3. NPD treated with Triovir (NPD + TRIO)
4. PD untreated control (PD)
5. PD treated with Truvada (PD + TRU)
6. PD treated with Triovir (PD + TRIO)

The NPD untreated group served as the normal control, whereas the PD untreated group served as the disease control. This grouping strategy was selected to assess the metabolic effects of cART in both normal and PD states and to distinguish the effects of prediabetes alone from those associated with ARV exposure.

### 2.5. Drug Preparation and Administration

Triovir is a fixed‐dose combination ARV tablet (1200 mg) containing TDF (300 mg), FTC (200 mg) and EFV (600 mg). It is prescribed for daily management of HIV infection. Truvada, another fixed‐dose ARV tablet (1000 mg), contains TDF (300 mg) and FTC (200 mg) and is used as daily PrEP to reduce the risk of contracting HIV. The human doses reported for both tablets apply to adults and adolescents weighing at least 35 kg. The animal equivalent doses (AED) were calculated following the United States Food and Drug Administration (U.S. FDA) guidance using the allometric scaling formula [22].

Animal equivalent dose AEDmgkg=Human equivalent dose mgkg x Km ratioKmhumanKmanimal.

The correction factor (K_m_) for humans was calculated by dividing the average body weight (35 kg) by the estimated body surface area (BSA) of 1.29 m^2^. BSA was estimated using the Du Bois formula based on an average weight of 35 kg and height of 160 cm. As a result, this yielded a human K_m_ factor of 27.13. For rats, a standardised K_m_ value of 6 was used. Applying the AED formula resulted in doses of approximately 128 mg/kg/day for Truvada and 194.8 mg/kg/day for Triovir in rats. Rats were weighed weekly, and individualised doses based on body weight were administered once daily by oral gavage between 0800 and 1000 hour for 12 weeks. Animals were closely monitored for 20 minutes postadministration for any adverse side effects; none were recorded. Food and water intake was not formally quantified during the study; however, no animals were lost as a direct result of the oral gavage procedure over the 12‐week treatment period.

### 2.6. Oral Glucose Tolerance Response

To evaluate glucose tolerance, the oral glucose tolerance test (OGTT) and area under the curve (AUC) were measured at Weeks 0 and 12 of the treatment phase following laboratory‐established protocol [23, 24]. Briefly, rats were fasted for 12 hours overnight; after which, glucose (0.86 g/kg, p.o.) was administered by oral gavage (18‐gauge, 38 mm long curved with a 21/4 mm ball end). Blood glucose was then monitored via tail prick using glucose strips (ACCU‐CHEK, Roche Diagnostics, United States) at 0, 15, 30, 60 and 120‐minute intervals postloading. A 12‐hour overnight fast was used for consistency with our group′s previously validated protocol [23, 24].

### 2.7. Terminal Blood and Tissue Collection

At the end of the treatment phase, animals were gently placed in a gas chamber (BRU, UKZN, Durban, South Africa) and anaesthetised with 100 mg/kg Isofor (Safeline Pharmaceuticals [Pty] Ltd., Roodepoort, South Africa) for approximately 3 minutes until unconscious. Blood was collected via cardiac puncture into EDTA‐coated tubes (The Scientific Group, Northriding, South Africa), gently inverted five times to ensure adequate anticoagulation and centrifuged (Eppendorf centrifuge 5403, LGBW Germany) at 503 × *g* for 15 minutes at 4°C to obtain plasma. Plasma was stored at −80°C in a Bio Ultra freezer (Snijers Scientific, Tilburg, Netherlands) until further biochemical analyses. The seven animals in a group were randomly selected at Phase 2 for biochemical and hormonal analyses that needed *n* = 5 animals per group to accommodate replication or duplicates in a 96‐well plates of the ELISA kit (because of financial constraints). However, *n* = 5 is normally acceptable in preclinical metabolic animal studies. This *n* = 5 per group is reflected in all results, figures and tables from Section 3.2 onward. Liver tissue samples were carefully excised, rinsed with cold normal saline solution, snap‐frozen in liquid nitrogen and stored at −80°C for subsequent analyses.

### 2.8. Homeostasis Model Assessment of Insulin Resistance and *β*‐Cell Function (HOMA‐IR and HOMA‐*β*)

The homeostasis model assessment (HOMA) is a robust surrogate method that utilises FPG and insulin concentrations to derive indices of insulin resistance (HOMA‐IR) and pancreatic *β*‐cell function (HOMA‐*β*) [25]. HOMA‐IR is based on the physiological feedback mechanism whereby blood glucose concentrations regulate insulin secretion from pancreatic *β*‐cells, providing an estimate of peripheral insulin resistance [26]. HOMA‐*β*, derived from the same fasting glucose and insulin values, instead reflects pancreatic *β*‐cell secretory capacity. Together, the two indices offer complementary insight into the distinct metabolic disturbances underlying impaired glucose regulation.

At the end of the treatment phase, insulin resistance and *β*‐cell function were estimated using the following formulas:

HOMA−IR=fasting plasma insulin μU/mL×fasting glucose mmol/L22.5,

HOMA−β=20×fasting insulin μU/mLfasting glucose mmol/L−3.5.

Briefly, fasting blood samples were collected via tail prick after 12 hours of overnight fasting. Blood glucose was measured using an ACCU‐CHEK instant system glucometer, and plasma insulin levels were determined using an enzyme‐linked immunosorbent assay (ELISA) kit (Cat No.: RK09268).

### 2.9. Biochemical Analysis

#### 2.9.1. ELISA

Key hormones involved in blood glucose regulation, including insulin (RK09268), glucagon (RK02835), C‐peptide (RK06829) and HbA1c (RK03710), were quantified using their respective ELISA kits, following the manufacturer′s instructions.

Plasma samples and standards were run in duplicates on 96‐well microplates. Plates were washed three times prior to use. After washing, 100 *μ*L of plasma samples and different concentrations of standards were added into the antibody precoated wells and incubated for 2 hours at 37°C. The wells were washed, and 100 *μ*L biotinylated detection antibody was incubated in the wells for 60 minutes at 37°C. Following repetition of the wash process, the wells were incubated with 100 *μ*L streptavidin‐horseradish peroxidase (HRP) conjugate for 30 minutes at 37°C. After a final wash, 90 *μ*L of 3,3 ^′^,5,5 ^′^ tetramethylbenzidine (TMB) substrate was added and incubated for 15–20 minutes at 37°C until a blue colour developed. The enzyme‐substrate reaction was terminated by adding 50 *μ*L of stop solution, and optical density (OD) was measured spectrophotometrically within 5 minutes at 450 nm with a correction wavelength at 570 nm.

#### 2.9.2. Hepatic Glucose Analysis

The terminally collected liver tissue samples were weighed and thereafter homogenised in PBS (tissue weight [g]: PBS [mL] volume = 1 : 9) using a mini‐handheld homogeniser (Hangzhou Miu Instruments Co., Ltd, Zhejiang, China). The homogenates were centrifuged at 5000 × *g* for 10 minutes at 5°C–8°C, and the resulting supernatants were collected for analysis. A well‐established colorimetric assay, the phenol‐sulphuric acid method, was used to quantify glucose [27]. To separate Eppendorf tubes, 100 *μ*L of glucose standards and liver tissue samples were added, in duplicate. To each tube, 300 *μ*L of concentrated sulphuric acid and 50 *μ*L of 5% phenol solution were added. To mix the contents, the tubes were gently vortexed for 5 seconds. Following thorough mixing, the tubes were boiled in a water bath at 90°C for 10 minutes, and then allowed to cool to room temperature; 250 *μ*L of the reaction mixture was then transferred onto a 96‐well plate and absorbance was measured at 490 nm using a spectrophotometer. Glucose concentrations were calculated using a standard curve generated from known concentrations of glucose, and results were expressed as mg glucose per gram of liver tissue.

### 2.10. Statistical Analysis

Data analysis and statistical calculations were performed using GraphPad Prism Version 7.0 (GraphPad Software Inc., San Diego, California, United States), presenting the data as mean ± standard error of the mean (SEM). Two‐way analysis of variance (ANOVA) was used to compare the data following treatment between subtreatment groups. Post hoc analysis was conducted using the Tukey honest significant difference test. Statistical significance was defined with a value of *p* < 0.05.

## 3. Results

### 3.1. Induction of Prediabetes and Its Diagnosis

At the end of the prediabetes induction phase, FPG, 2‐hr PG, body weight and triglycerides were measured between the NPD and PD groups (Table 1). Using the American Diabetes Association (ADA) diagnostic thresholds, the PD animals demonstrated FPG levels within the IFG range (6.3 ± 0.381 mmol/L), whereas the NPD group remained below the cutoff (4.457 ± 0.232 mmol/L). The PD group met ADA criteria for prediabetes on the basis of FPG, without meeting the IGT threshold on 2‐hour OGTT. In addition to dysglycemia, PD animals exhibited significantly higher body weight and elevated triglyceride concentrations (*p* < 0.05, Table 1).

### 3.2. The Effect of cART Regimens on Body Weight

Body weights were measured in the NPD‐ and PD‐treated groups at the end of the treatment period. Results showed that under normal conditions, in the NPD group, there were no statistically significant differences in body weight among the control, Truvada‐ and Triovir‐treated animals. In contrast, under **PD** conditions, in the PD group, the control animals exhibited a significant increase in body weight compared with the NPD control group (*p* < 0.05, Figure 2). Treatment with Truvada and Triovir in PD animals resulted in slightly lower body weights compared with the PD control group; however, these differences were not statistically significant. Interestingly, the Truvada‐treated PD group demonstrated a significant increase in body weight compared with the Truvada‐treated NPD group (*p* < 0.05, Figure 2).

**Figure 2:**
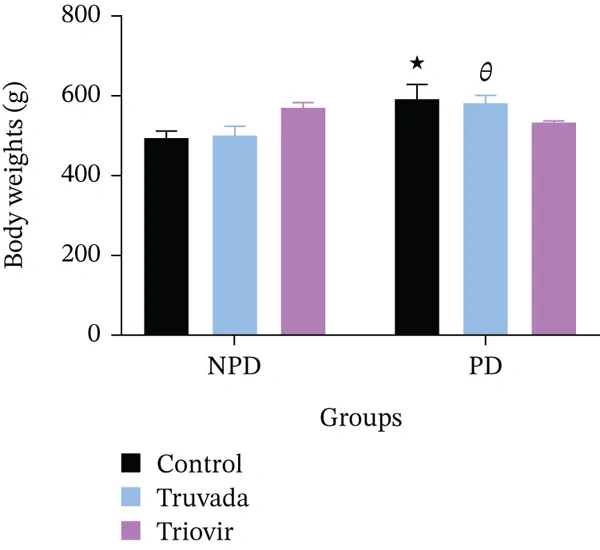
The effects of Truvada and Triovir on body weight in nonprediabetic (NPD) and prediabetic (PD) groups (*n* = 5 per group) at the end of the experimental period. The results are expressed as mean ± SEM. ★ = *p* < 0.05 denotes comparison with NPD; *θ* = *p* < 0.05 denotes significance in comparison to the Truvada‐treated NPD group.

### 3.3. The Effect of cART Regimens on Plasma Lipid Profiles

Plasma triglycerides and total cholesterol were assessed as indices of lipid metabolism; HDL‐ and LDL‐C formed the part of the vascular study results. In the NPD group, there were no statistically significant differences in triglyceride levels among the control, Truvada‐ and Triovir‐treated animals (*p* > 0.05, Figure 3A,B). Conversely, in the PD group, a significant increase in triglyceride levels was observed in control animals compared with the NPD control group (*p* < 0.05, Figure 3A). Treatment with Truvada in PD animals significantly reduced triglyceride levels compared with the PD control (*p* < 0.05, Figure 3A), whereas Triovir treatment resulted in a modest but non‐significant reduction in triglyceride levels relative to PD control (*p* > 0.05). In contrast, analysis of total cholesterol levels (Figure 3B) revealed no statistically significant differences between treatment groups within either metabolic condition.

**Figure 3:**
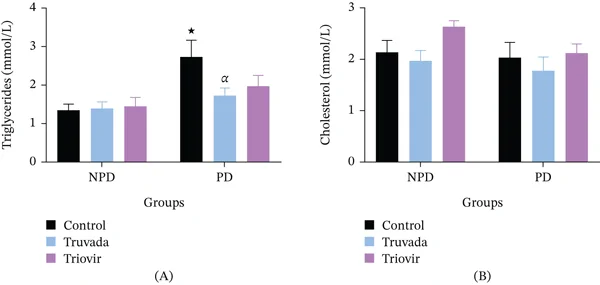
The effects of Truvada and Triovir on (A) plasma triglyceride and (B) total cholesterol concentrations in nonprediabetic (NPD) and prediabetic (PD) groups (*n* = 5 per group). Results are expressed as mean ± SEM. ★ = *p* < 0.05 denotes comparison with NPD; *α* = *p* < 0.05 denotes comparison with PD.

### 3.4. The Effect of cART Regimens on Oral Glucose Tolerance Response

To assess the effects of Truvada and Triovir on glucose metabolism, FPG and 2‐hour postglucose load concentrations (Table 2) were measured from OGTTs performed at the end of the treatment period in NPD and PD groups, and the AUC was subsequently calculated (Figure 4).

**Table 2:** Fasting plasma glucose and 2‐hour plasma glucose concentrations in the nonprediabetic (NPD) and prediabetic (PD) cART‐treated groups following an oral glucose load (0.86 g/kg, p.o.).

	NPD			PD		
	Control	Truvada	Triovir	Control	Truvada	Triovir
Fasting plasma glucose	4.72 ± 0.15	4.83 ± 0.12	5.09 ± 0.11	5.51 ± 0.13^∗^	5.4 ± 0.10 *θ*	5.7 ± 0.09 *δ*
2‐hour plasma glucose	4.76 ± 0.24	4.44 ± 0.19	4.31 ± 0.05	7.27 ± 0.19^∗^	5.9 ± 0.19 *αθ*	5.59 ± 0.19 *αδ*

*Note:* Values are presented as means ± SEM (*n* = 5 per group). ^∗^ (*p* < 0.05) vs. NPD; *α* (*p* < 0.05) vs. PD; *θ* (*p* < 0.05) vs. Truvada‐treated NPD group; 𝛿 (*p* < 0.05) vs. Triovir‐treated NPD group.

**Figure 4:**
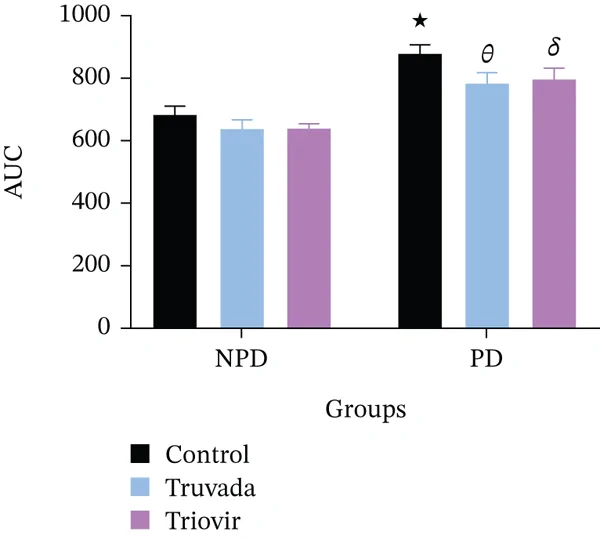
Area under the curve in nonprediabetic (NPD) and prediabetic (PD) cART‐treated groups (*n* = 5 per group) at Week 12 of treatment. Results are expressed as mean ± SEM. Statistical significance is indicated by ★ (*p* < 0.05) vs. NPD; *θ* (*p* < 0.05) vs. Truvada‐treated NPD group; 𝛿 (*p* < 0.05) vs. Triovir‐treated NPD group.

In the NPD groups, fasting glucose levels remained within the normal range, with no significant differences observed among the control, Truvada‐ and Triovir‐treated animals (*p* > 0.05; Table 2). Similarly, 2‐hour glucose concentrations and glucose AUC were not significantly changed across all treatment groups (*p* > 0.05, Table 2 and Figure 4).

In contrast, the PD control group exhibited significant impairments in glucose regulation, with significant increases in FPG, 2‐hour glucose concentrations and AUC values compared with the NPD control group (*p* < 0.05). Treatment with Truvada in PD animals resulted in a modest, non‐significant decrease in fasting glucose, whereas Triovir treatment caused a slight, non‐significant increase compared with the PD control group (*p* > 0.05). Both treatments, however, significantly reduced 2‐hour glucose levels relative to the PD control group (*p* < 0.05). Despite these effects, FPG, 2‐hour glucose and AUC values in Truvada‐treated PD animals remained significantly higher than those observed in Truvada‐treated NPD animals. Similarly, these parameters in Triovir‐treated PD animals were significantly elevated compared with Triovir‐treated NPD animals (*p* < 0.05).

### 3.5. The Effect of cART Regimens on HbA1c

In the NPD groups, both Truvada‐ and Triovir‐treated animals exhibited significantly higher HbA1c levels compared with the NPD control group (*p* < 0.05, Figure 5). No significant differences were observed between the NPD and PD control groups (*p* > 0.05, Figure 5). Within the PD groups, Truvada‐treated animals showed a non‐significant increase in HbA1c levels compared with the PD control group. Furthermore, Triovir‐treated PD animals displayed significantly lower HbA1c levels compared with both Truvada‐treated PD and Triovir‐treated NPD animals (*p* < 0.05, Figure 5).

**Figure 5:**
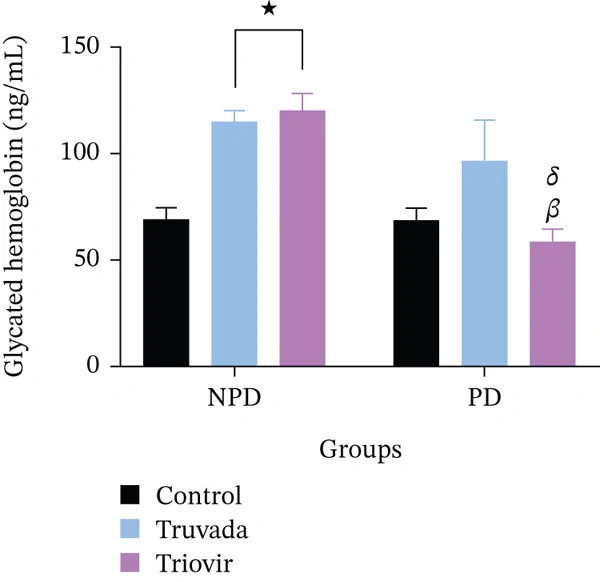
The effects of Truvada and Triovir on HbA1c in nonprediabetic (NPD) and prediabetic (PD) groups (*n* = 5 per group). Results are expressed as mean ± SEM. Statistical significance is indicated by ★ (*p* < 0.05) vs. NPD; *β* (*p* < 0.05) Truvada vs. Triovir; 𝛿 (*p* < 0.05) vs. Triovir‐treated NPD group.

### 3.6. The Effect of cART Regimens on Glucoregulatory Hormones

Truvada‐treated NPD group had significantly elevated insulin levels compared with the NPD control group (*p* < 0.05, Figure 6A). In contrast, Triovir treatment in the NPD group showed a non‐significant decrease in plasma insulin levels compared with the NPD control (*p* > 0.05, Figure 6A). Plasma insulin levels were significantly lower in Triovir‐treated animals compared with those treated with Truvada (*p* < 0.05, Figure 6A). In the PD group, plasma insulin levels in the PD control were significantly higher than in the NPD control (*p* < 0.05, Figure 6A). Both Truvada and Triovir treatments decreased plasma insulin levels compared with the PD control (*p* < 0.05, Figure 6A), with Triovir‐treated PD animals exhibiting significantly lower plasma insulin than the Truvada‐treated PD group (*p* < 0.05, Figure 6A).

**Figure 6:**
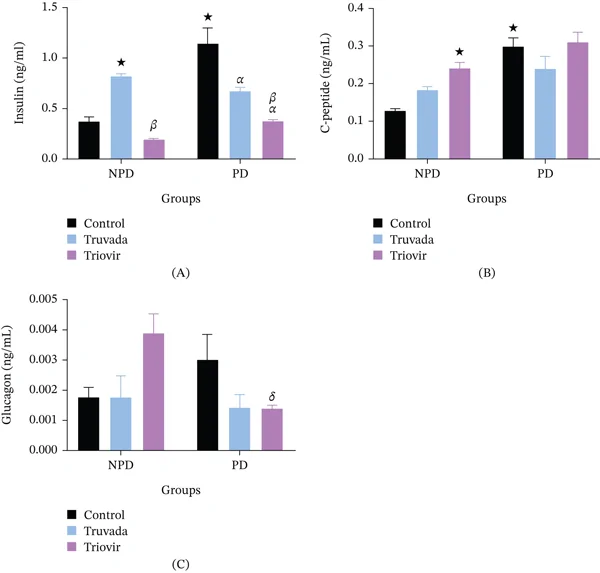
The effects of Truvada and Triovir on terminal plasma (A) insulin, (B) C‐peptide and (C) glucagon concentrations in the nonprediabetic (NPD) and prediabetic (PD) groups (*n* = 5 per group). Results are expressed as mean ± SEM. Statistical significance is indicated by ★ (*p* < 0.05) vs. NPD; *α* (*p* < 0.05) vs. PD; *β* (*p* < 0.05) Truvada vs. Triovir; 𝛿 (*p* < 0.05) vs. Triovir‐treated NPD group.

Plasma C‐peptide levels were elevated in both Truvada‐ and Triovir‐treated NPD groups compared with the NPD control, with statistical significance observed only in the Triovir‐treated group (*p* < 0.05, Figure 6B). The PD control group similarly exhibited significantly higher C‐peptide levels than the NPD control group (*p* < 0.05, Figure 6B). However, no significant differences were observed between treatment groups within the PD state (*p* > 0.05, Figure 6B).

Plasma glucagon analysis showed that in the NPD group, Truvada treatment resulted in glucagon levels similar to the NPD control, whereas Triovir treatment caused an increase in glucagon levels; however, none of these changes were statistically significant (*p* > 0.05, Figure 6C). In the PD state, both Truvada‐ and Triovir‐treated PD animals exhibited reduced glucagon levels compared with the PD control, although these differences were not significant (*p* > 0.05, Figure 6C). Additionally, the Triovir‐treated PD group had significantly lower glucagon levels compared with the Triovir‐treated PD group (*p* < 0.05, Figure 6C).

### 3.7. The Effect of cART Regimens on Homeostasis Model Assessment (HOMA)

At the end of the treatment phase, HOMA‐IR and HOMA‐*β* were calculated to assess the effects of Truvada and Triovir on insulin resistance (Figure 7A) and pancreatic *β*‐cell function (Figure 7B), respectively. The Truvada‐treated animals demonstrated significantly higher HOMA‐IR values compared with the NPD control (*p* < 0.05, Figure 7A). In contrast, Triovir‐treated NPD animals exhibited a non‐significant reduction in HOMA‐IR compared with the NPD control group (*p* > 0.05, Figure 7A). Moreover, HOMA‐IR values were significantly lower in Triovir‐treated NPD animals compared with those treated with Truvada (*p* < 0.05, Figure 7A). In the PD group, control animals exhibited a significant increase in HOMA‐IR compared with the NPD control (*p* < 0.05, Figure 7A). Both Truvada and Triovir treatments in PD animals significantly reduced HOMA‐IR levels relative to the PD control group (*p* < 0.05, Figure 7A).

**Figure 7:**
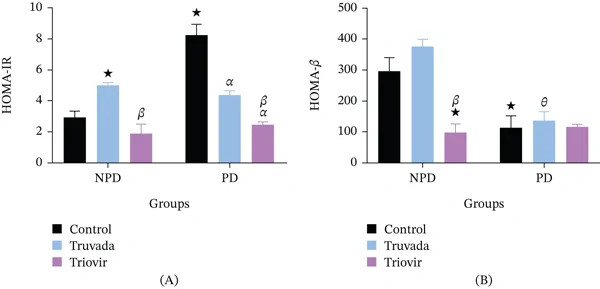
The effects of Truvada and Triovir on (A) insulin resistance and (B) pancreatic *β*‐cell function in the nonprediabetic (NPD) and prediabetic (PD) groups (*n* = 5 per group). Results are expressed as mean ± SEM. Statistical significance is indicated by ★ (*p* < 0.05) vs. NPD; *α* (*p* < 0.05) vs. PD; *β* (*p* < 0.05) Truvada vs. Triovir; *θ* (*p* < 0.05) vs. Truvada‐treated NPD group.

Pancreatic *β*‐cell function findings, as illustrated in Graph 7B (HOMA‐*β*), indicate that within the NPD groups, Truvada‐treated animals showed a non‐significant increase in HOMA‐*β* values compared with the NPD control (*p* > 0.05, Figure 7B). Conversely, Triovir‐treated NPD animals exhibited significantly lower HOMA‐*β* values than both the NPD control (*p* < 0.05, Figure 7B) and Truvada‐treated NPD groups (*p* < 0.0001). In the PD group, control animals displayed significantly lower HOMA‐*β* values compared with the NPD control group (*p* < 0.05, Figure 7B). Both Truvada and Triovir treatments in PD animals led to non‐significant changes in HOMA‐*β* relative to the PD control group (*p* > 0.05, Figure 7B). Moreover, the Truvada‐treated PD group showed a significant reduction in HOMA‐*β* compared with the Truvada‐treated NPD group (*p* < 0.0001).

### 3.8. The Effect of cART on Hepatic Glucose

Hepatic glucose concentration was assessed to further evaluate the effects of Truvada and Triovir on glucose metabolism in the NPD and PD groups (Figure 8). In the NPD groups, both Truvada**-** and Triovir‐treated animals exhibited significantly lower hepatic glucose concentrations compared with the NPD control group (*p* < 0.05, Figure 8). Among the PD groups, control animals also showed a significant reduction in hepatic glucose levels relative to the NPD control group (*p* < 0.05, Figure 8). Treatment with Truvada in PD animals resulted in a significant increase in hepatic glucose compared with both the PD control and the Truvada‐treated NPD group (*p* < 0.05, Figure 8). Triovir administration in PD animals resulted in a modest non‐significant increase in hepatic glucose levels relative to the PD control group (*p* > 0.05, Figure 8).

**Figure 8:**
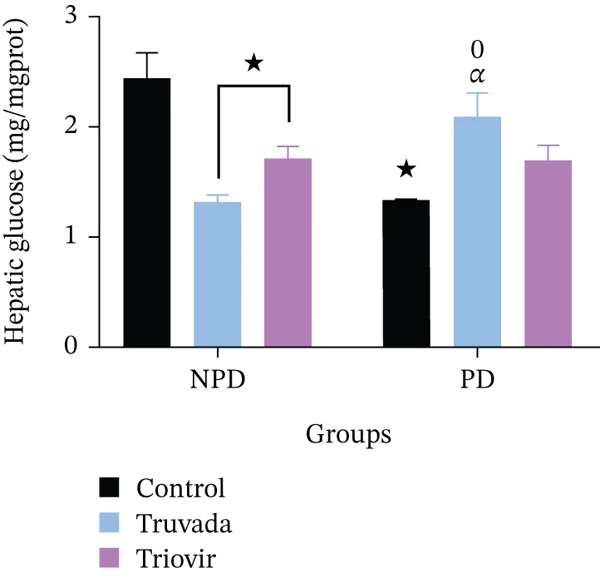
The effects of Truvada and Triovir on hepatic glucose in the nonprediabetic (NPD) and prediabetic (PD) groups (*n* = 5 per group). Results are expressed as mean ± SEM. Statistical significance is indicated by the following: ★ = *p* < 0.05 denotes comparison with NPD; *α* = *p* < 0.05 denotes comparison with PD; *θ* = *p* < 0.05 denotes significance in comparison to the Truvada‐treated NPD group.

### 3.9. Correlation Analysis

The correlation between body weight and triglycerides, FPG and HbA1c, as well as glucagon and hepatic glucose was assessed across all animals and within each treatment group (Tables 3). Data were initially evaluated for normality using the Shapiro–Wilk test. Pearson′s correlation coefficients (*r*) were applied when both variables were normally distributed, whereas Spearman rank correlation coefficients (*p*) were used when either variable violated normality assumptions, providing a robust non‐parametric alternative [28]. Overall correlations across all animals were weak or negligible; however, subgroup analyses revealed more distinct patterns.

**Table 3:** Correlation analyses between body weight and triglycerides, FPG and HbA1c and glucagon and hepatic glucose across all animals and within treatment groups.

**Body weight (g) vs. triglycerides (mmol/L)**			
	**Group**	**Spearman correlation (** **p** **)**	**Strength of association**
Overall	All animals	0.1277	Weak
Treatment groups	NPD Control	0	Negligible
	NPD Truvada	0.4	Moderate
	NPD Triovir	0.4	Moderate
	PD Control	−0.4	Moderate
	PD Truvada	−0.8	Strong
	PD Triovir	0.2	Weak

**Fasting plasma glucose (mmol/L) vs. HbA1c (ng/mL)**			

	**Group**	**Pearson Correlation (** **r** **)**	**Strength of association**
Overall	All animals	−0.3891	Weak
Treatment groups	NPD Control	−0.35	Weak
	NPD Truvada	0.3423	Weak
	NPD Triovir	−0.5526	Moderate
	PD Control	0.2078	Weak
	PD Truvada	−0.2669	Weak
	PD Triovir	‐0.157	Weak

**Glucagon (ng/mL) vs. hepatic glucose (mg/mgprot)**			

	**Group**	**Spearman correlation (** **p** **)**	**Strength of association**
Overall	All animals	0.0561	Negligible
Treatment groups	NPD Control	0.4	Moderate
	NPD Truvada	0.4	Moderate
	NPD Triovir	0.8	Strong
	PD Control	0.6	Moderate
	PD Truvada	1	Very strong
	PD Triovir	0.8	Strong

## 4. Discussion

The increasing prevalence of T2DM risk factors and the rising rates of prediabetes among PLHIV have raised concerns regarding the long‐term metabolic consequences of current antiretroviral therapy (ART) regimens [29]. Although metabolic dysfunctions have traditionally been associated with lifestyle and behavioural factors, accumulating evidence indicates that chronic ART exposure can also disrupt lipid and glucose homeostasis [30]. However, the specific effects of NtRTI/NRTI‐ and NNRTI‐based therapies on glucose homeostasis remain underexplored, particularly in the context of prediabetes.

In this study, we investigated the metabolic effects of two fixed‐dose combination ARV drugs, TDF/FTC (Truvada) and TDF/FTC/EFV (Triovir), commonly used for HIV prevention and treatment, respectively. Truvada is an NtRTI/NRTI combination containing TDF and FTC, whereas Triovir includes an additional NNRTI, EFV. Using a diet‐induced PD rat model, the study evaluated how these cART regimens affect metabolic parameters under both normal and PD conditions. The overarching aim of the study was to investigate whether these cART regimens might predispose metabolically healthy individuals to prediabetes with sustained exposure. Concurrently, the study also examined whether the same regimens could protect PD individuals from progressing to overt T2DM.

Prediabetes is a multifactorial and unstable condition that is challenging to diagnose because of fluctuations in metabolic markers [19]. Findings showed that at the end of the 20‐week induction phase, animals in the PD group showed elevated FPG, indicating IFG, along with significantly increased body weight and triglyceride levels compared with NPD controls, confirming the successful establishment of a PD phenotype. Interestingly, there was no significant difference in 2‐hr PG levels between the PD control and NPD control groups, suggesting that overt glucose intolerance had not yet developed, likely because of the activation of compensatory mechanisms such as hyperinsulinaemia. Traditionally, rapid body weight gain is associated with hyperlipidaemia and adipose tissue dysfunction [31]. In this study, PD control animals exhibited increased body weight and elevated triglyceride levels compared with NPD control animals. This may be attributed to the different diets to which the groups were exposed, namely, the HFHC diet and standard rat chow, respectively. Accordingly, the greater body weight and hypertriglyceridaemia observed in the PD control animals are consistent with the known effects of a HFHC diet, which promotes sustained positive energy balance, adipose tissue expansion and lipid dysregulation [32]. In PD animals, Truvada did not alter total body weight gain, whereas Triovir non‐significantly reduced weight gain. This reduction may reflect the known weight‐suppressing effects of EFV, which has been linked to appetite suppression [33–35]. However, these observations come from HIV‐positive patients, where chronic inflammation due to viral infection also contributes to weight reduction. The observation of reduced weight gain in PD animals, despite the absence of HIV‐mimicking proteins and thus HIV‐related inflammation, supports the presence of underlying low‐grade inflammation inherent to prediabetes [36–38]. In contrast, Triovir produces a non‐significant increase in body weight in NPD animals, supporting the interpretation that the attenuated weight gain observed in PD rats treated with Triovir may stem from pre‐existing low‐grade inflammation rather than EFV exposure alone.

Alterations in body weight were reflected in lipid metabolism, particularly in PD animals. PD controls exhibited elevated plasma triglycerides with no corresponding change in total cholesterol. Truvada treatment lowered both total cholesterol and triglycerides in PD and NPD rats, with its reported lipid‐lowering properties and with the reduced weight gain observed in Triovir‐treated PD rats. The reduction in triglycerides in Truvada‐treated PD animals, despite the absence of EFV, suggests that TDF, FTC or their combination (TDF/FTC) may exert intrinsic lipid‐lowering effects, potentially through TDF‐mediated regulation of hepatic CD36/PPAR‐*α* activity [39–41].

In contrast, Triovir produced a metabolically divergent profile. In NPD rats, Triovir treatment caused a slight increase in total plasma cholesterol without altering plasma triglyceride levels. This lipid profile pattern resembles diabetic dyslipidaemia, where elevations in low‐density lipoprotein cholesterol (LDL‐C) contribute to hypercholesterolemia, a well‐established risk factor for cardiovascular disease [42]. The concurrent increase in body weight and total cholesterol in NPD animals contrasts with previous reports associating TDF‐ and EFV‐containing regimens with weight gain suppression and normolipidaemic profiles, suggesting that such effects are likely influenced by virus‐induced low‐grade inflammation rather than drug exposure alone. EFV has been reported to promote AMPK‐driven *β*‐oxidation, increasing cellular acetyl‐CoA availability, a precursor for cholesterol biosynthesis via the mevalonate pathway, which may explain the observed cholesterol elevation even under normal metabolic conditions [43]. In contrast, PD animals treated with Triovir exhibited reduced plasma triglycerides with no change in total cholesterol. This effect may be attributed to the lower body weight gain observed in these animals and the previously reported metabolic effects of EFV, including concentration‐dependent mitochondrial toxicity, impaired adipocyte differentiation and neuropsychiatric effects that can alter appetite and energy balance [35]. Additionally, PD conditions may be associated with impaired hepatic function which leads to elevated EFV concentrations because of altered cytochrome P450 2B6 activity, further modulating its metabolic impact in PD animals [44]. Therefore, assessing the pharmacokinetic parameters of EFV in prediabetes can be invaluable in providing further insight into the current observations.

These alterations in lipid metabolism corresponded with changes in glucose regulation, consistent with the well‐established relationship between impaired lipid metabolism and disrupted glucose homeostasis [45]. PD control animals with elevated triglycerides also exhibited increased FPG and OGTT AUC, whereas 2‐hr PG remained comparable with NPD controls. Truvada treatment had no effect on FPG or OGTT AUC in either metabolic state; however, it significantly reduced 2‐hr PG in PD animals, suggesting modest improvements in glucose tolerance, possibly through enhanced skeletal muscle glucose uptake and utilisation or improved insulin‐signalling pathways. Furthermore, Triovir treatment produced modest effects on glucose homeostasis that appeared largely independent of metabolic state. In both NPD and PD animals, FPG increased slightly, whereas 2‐hr PG and OGTT AUC decreased post‐treatment. In NPD animals, this profile may indicate emerging drug‐induced metabolic perturbations, whereas in PD animals, the same pattern likely reflects compensatory mechanisms activated in response to chronic exposure to a high‐calorie diet.

HbA1c reflects long‐term glycaemic control because it represents average blood glucose over the 120‐day lifespan of red blood cells, making it less sensitive to the short‐term fluctuations typical of early prediabetes [18, 46, 47]. In this study, HbA1c levels did not differ significantly between NPD and PD control groups despite higher glucose levels in PD rats, a finding that contrasts with previous reports [48, 49]. To the best of our knowledge, there are no published animal studies that directly assess HbA1c responses to TDF‐ or EFV‐based ARV regimens in PD models. In our study, PD animals treated with Truvada (TDF/FTC) showed increased HbA1c, whereas Triovir (TDF/FTC/EFV) produced a reduction. The rise observed with TDF/FTC may reflect the underlying insulin resistance characteristic of the PD state, whereas the decrease associated with EFV‐containing regimen suggests that EFV may modulate glucose handling or protein glycation differently in an insulin‐resistant state. In NPD animals, both regimens increased HbA1c, indicating that ART may enhance protein glycosylation. Although TDF/FTC is generally associated with favourable metabolic effects, these findings raise the possibility of ART‐related alterations in insulin structure or insulin‐receptor interactions, warranting further investigation into ART‐driven effects on protein glycation.

This study also assessed HOMA‐IR and HOMA‐*β* as indices of systemic insulin resistance and *β*‐cell function, respectively. PD controls showed elevated HOMA‐IR and reduced HOMA‐*β*, indicating that insulin resistance and *β*‐cell dysfunction emerge early in prediabetes, preceding overt glycemic dysregulation [50]. These findings corroborate prior reports showing that diet‐induced prediabetes triggers early disruptions in glucose homeostasis [24, 49, 51]. Administration of Truvada and Triovir treatments in PD animals improved insulin sensitivity without significantly altering *β*‐cell function. This pattern suggests that the observed improvements arise from enhanced responsiveness of target tissues to insulin rather than increased insulin secretion. In contrast, under normal metabolic conditions, both regimens produced distinct and unexpected effects. Truvada increased both HOMA‐IR and HOMA‐*β* in NPD animals, indicating reduced insulin sensitivity accompanied by a compensatory increase in pancreatic insulin secretion. This pattern reflects an early‐stage compensatory mechanism activated to maintain glucose homeostasis in response to emerging metabolic dysfunction. Conversely, Triovir decreased both HOMA‐IR and HOMA‐*β* in NPD animals. Although the reduction in HOMA‐IR suggests improved insulin sensitivity, the concurrent decline in *β*‐cell function indicates reduced insulin secretion. This combination may indicate a lower insulin demand due to enhanced target tissue responsiveness; however, it could also suggest modest *β*‐cell suppression, highlighting a complex effect of Triovir on glucose regulation in NPD animals.

Furthermore, HOMA‐IR serves as a model for assessing the effectiveness of secreted insulin in facilitating glucose uptake and lowering blood glucose. Elevated FPG, accompanied by increased circulating insulin and high HOMA‐IR, indicates both insulin resistance and compensatory hyperinsulinemia, which can further exacerbate metabolic dysfunction [52, 53]. In this study, PD controls exhibited hyperinsulinemia along with elevated plasma C‐peptide, indicating compensatory *β*‐cell hypersecretion in response to insulin resistance. Post‐treatment with both Truvada and Triovir in PD animals, plasma insulin levels were significantly reduced, whereas C‐peptide remained elevated across both treatment groups. This may be attributed to insulin resistance‐induced compensatory mechanisms, leading to increases in insulin secretion. In Truvada‐treated PD animals, the reduction of circulating insulin corresponded with a slight reduction in C‐peptide, signifying improved insulin sensitivity, consistent with the reduced HOMA‐IR observed and an effect commonly associated with TDF. However, in Triovir‐treated PD animals, the marked reduction in insulin was not accompanied by a reduction in C‐peptide, suggesting a potential EFV‐insulin interaction affecting insulin stability or clearance. Hence, insulin resistance traditionally associated with EFV‐containing regimens may not stem solely from impaired insulin signalling in target organs but could also involve allosteric effects on insulin itself. Moreover, treatment with Truvada in NPD animals increased plasma insulin and C‐peptide, whereas Triovir produced low insulin but high C‐peptide levels, suggesting that insulin is being secreted but not appearing in circulation, further implying altered insulin bioavailability in the presence of EFV.

Under normal physiological conditions, glucose homeostasis is regulated by the opposing actions of insulin and glucagon [3]. In this study, PD control animals displayed elevated glucagon levels, and their concurrent hyperinsulinaemia and hyperglucagonemia suggest pancreatic dysfunction secondary to insulin resistance [54, 55]. In PD animals, both EFV‐containing (Triovir) and non‐EFV‐containing (Truvada) lowered glucagon levels. In contrast, Triovir increased glucagon in NPD rats, possibly because of pancreatic *α*‐cell responses to elevated FPG arising from systemic insulin resistance.

Glucagon promotes hepatic glucose production through glycogenolysis and gluconeogenesis [17, 56]. In this study, PD control animals showed reduced hepatic glucose, likely reflecting hyperinsulinaemia‐driven activation of hepatic glycogen synthase, with concurrent hyperglycaemia providing substrate to maximise glycogen storage [57], an effect previously noted to precede hepatic insulin resistance. Following treatment, both Truvada‐ and Triovir‐treated PD animals exhibited increased hepatic glucose concentrations and elevated HOMA‐IR, consistent with worsening systemic insulin resistance, subsequent hepatic insulin resistance and impaired glycogen synthase activity. Furthermore, drug‐induced hepatic metabolic dysfunction and the hepatic accumulation of ARV metabolites in the liver may also contribute to this effect [58].

In NPD animals, both cART regimens reduced hepatic glucose, suggesting that Truvada and Triovir may alter hepatic glucose handling, even in normal metabolic states. For Truvada, this reduction may reflect hyperinsulinaemia‐driven effects on hepatic glucose regulation. For EFV‐containing Triovir, the underlying mechanism remains unclear, and further studies are needed to investigate potential interactions of EFV with hepatic glycogen synthase under normal physiological conditions.

## 5. Conclusion

Reducing progression from prediabetes to T2DM is essential for advancing Sustainable Development Goal (SDG) 3, especially for PLHIV, who face a substantial risk of developing T2DM than the general population. This study demonstrates that Truvada and Triovir exert distinct metabolic state‐dependent effects on glucose homeostasis. In metabolically healthy (NPD) animals, Truvada increased circulating insulin and HbA1c, elevated HOMA‐IR and suppressed hepatic glucose output, features that are consistent with emerging insulin resistance with compensatory hyperinsulinaemia. These findings suggest Truvada may initiate early glycaemic disturbances and increase long‐term metabolic risk. Triovir, in contrast, reduced HOMA‐*β*, increased HbA1c and suppressed hepatic glucose output while preserving *β*‐cell insulin release and modestly improving peripheral insulin sensitivity. This profile indicates pancreatic *β*‐cell strain but less pronounced early insulin resistance, underscoring the complexity of EFV‐containing regimens in metabolically normal states. In PD animals, both drugs reduced compensatory hyperinsulinaemia, postprandial glucose and HOMA‐IR, suggesting short‐term metabolic improvements in an already impaired and fluctuating state. However, hepatic glucose output remained elevated, particularly with Truvada, indicating persistent hepatic glucose dysregulation that may increase the risk of long‐term dysglycaemia and progression to T2DM. Overall, these findings demonstrate the importance of individualised ART selection and regular metabolic monitoring to preserve metabolic health and reduce long‐term cardiometabolic risk, particularly in individuals with pre‐existing metabolic dysregulation.

### 5.1. Study Limitations

There a few limitations associated with this study which include the gender or sex representation in the animals; only male rats were used, consistent with established model of prediabetes and to avoid oestrous‐cycle‐related metabolic variability. However, given the well‐documented sex differences in cardiometabolic disease, including the insulin‐sensitising and anti‐inflammatory effects of oestrogen and sex‐specific patterns of fat distribution, our findings cannot be assumed to generalise to female physiology, and sex‐comparative studies are needed. The use of *n* = 5 in ELISA experiments also reduces the statistical power to detect the pharmacological effects. Moreover, our lipid panel was limited to triglycerides and total cholesterol; the HDL‐ and LDL‐C fractionation was already a part of the vascular study from the same rat cohort. The study design did not include the metabolic effect of the HIV infection, whereas in PLHIV, cART is administered against a background of chronic immune activation and systemic inflammation that may independently affect glucose and lipid metabolism and interact with drug effects in ways this model cannot capture.

Future studies should also examine the role of glucagon‐like peptide‐1 (GLP‐1), which enhances glucose‐stimulated insulin secretion, slows gastric emptying and suppresses food intake, particularly in individuals with pre‐existing metabolic dysfunction resulting from chronic consumption of high‐calorie diets. Additionally, evaluating inflammation and insulin‐signalling pathways could clarify how ART affects metabolic regulation across different metabolic states. Furthermore, studies using a combined HIV‐transgenic or HIV‐protein‐exposed PD model are needed to determine whether the present findings hold in the presence of the virus and increase the number of animals per groups even during the biochemical analysis.

## Funding

This study was supported by the National Research Foundation (10.13039/501100001321, TTK220324671), the South African Medical Research Council (10.13039/501100001322), Self‐Initiated Research Grant (SAMRC‐SIR).

## Conflicts of Interest

The authors declare no conflicts of interest.

## Data Availability

The data that support the findings of this study are available on request from the corresponding author. The data are not publicly available due to privacy or ethical restrictions.
